# An Efficient Method to Prepare Barcoded cDNA Libraries from Plant Callus for Long-Read Sequencing

**DOI:** 10.3390/mps6020031

**Published:** 2023-03-15

**Authors:** Daniela Cordeiro, Alexandra Camelo, Ana Carolina Pedrosa, Inês Brandão, Jorge Canhoto, Christophe Espírito Santo, Sandra Correia

**Affiliations:** 1 Centre for Functional Ecology, TERRA Associate Laboratory, Department of Life Sciences, University of Coimbra, Calçada Martim de Freitas, 3000-456 Coimbra, Portugal; 2 Centro de Apoio Tecnológico Agro-Alimentar (CATAA) de Castelo Branco, 6000-459 Castelo Branco, Portugal; 3 InnovPlantProtect CoLab, Estrada de Gil Vaz, 7350-478 Elvas, Portugal

**Keywords:** amplification-free protocol, direct cDNA sequencing, high-throughput sequencing, MinION, Oxford Nanopore Technologies^®^, poly(A) RNA, Solanaceae, *Solanum betaceum*, transcriptome, woody plant

## Abstract

Long-read sequencing methods allow a comprehensive analysis of transcriptomes in identifying full-length transcripts. This revolutionary method represents a considerable breakthrough for non-model species since it allows enhanced gene annotation and gene expression studies when compared to former sequencing methods. However, woody plant tissues are challenging to the successful preparation of cDNA libraries, thus, impairing further cutting-edge sequencing analyses. Here, a detailed protocol for preparing cDNA libraries suitable for high throughput RNA sequencing using Oxford Nanopore Technologies^®^ is described. This method was used to prepare eight barcoded cDNA libraries from two *Solanum betaceum* cell lines: one with compact morphology and embryogenic competency (EC) and another with friable and non-embryogenic (NEC). The libraries were successfully sequenced, and data quality assessment showed high mean quality scores. Using this method, long-read sequencing will allow a comprehensive analysis of plant transcriptomes.

## 1. Introduction

In recent years, transcriptome sequencing has been an invaluable tool in gene expression profiling. By unraveling gene regulatory mechanisms, high-throughput sequencing approaches have shown widespread applications in many fields of biology, including plant physiology and developmental analysis [1,2,3].

Nanopore sequencing from Oxford Nanopore Technologies^®^ (ONT) is a high-throughput sequencing technology that detects ionic current changes as DNA or RNA molecules pass through nanopores [4]. Compared to other next-generation sequencing platforms, Nanopore offers native and long-read sequencing (>4 Mb in a single read), which together with the template-switching method, allows the detection of complete full-length transcripts [5]. Such approaches, allowing for the complete reading of genomic information, are extremely important for non-sequenced species, which contributed to highlighting long-read sequencing as the Method of the Year 2022 in Nature [6]. Moreover, although there are no commercial options available for RNA, barcoding kits are available in Nanopore, which allows for cDNA tagging. Therefore, multiple barcoded libraries can be pooled and sequenced in a single run (using one flow cell), optimizing the assay for high throughput, and reducing the cost per sample [7]. With Nanopore, it is also possible to sequence cDNA directly using a PCR-free protocol, thus, avoiding amplification bias, and preserving the quantitative information of the samples [8].

Despite the rapid advances in the development and optimization of sequencing technologies, some methods are not so straightforwardly applicable to particular organisms. Due to the richness of secondary metabolites and high ribonuclease (RNase) contents, plant tissues have proven to be a complicated challenge for the application of cutting-edge sequencing technologies and obtaining high-quality RNA yields for the subsequent construction of good-quality cDNA libraries [9]. Although several kits are available for total RNA extraction and poly(A) RNA isolation, they have proven to be poorly effective for woody plant tissues, which makes it difficult to reproduce the results in different laboratories, which is a limitation in gene expression studies, particularly in non-model species [10,11]. Furthermore, RNA extraction yields vary widely according to species, sample type, genotypes, age, and oxidative state [10,11,12,13]. For instance, within in vitro grown calluses rich in starch, while pectins, among other polysaccharides (e.g., arabinoxylan, β-glucan) [14,15], have proven to be a particularly difficult tissue from which to achieve high yields and quality RNA extractions for further processing methods [10]. 

Owing to their great morphogenic plasticity, callus samples are valuable experimental models for fundamental research, as well as for industrial and bioengineering applications [16]. However, straightforward optimized procedures for high-throughput analyses are still often required. In this work, barcoded cDNA libraries were prepared from *Solanum betaceum* Cav. callus lines. *S. betaceum* is a solanaceous fruit tree, commonly known as tamarillo or tree tomato, which can be induced in vitro, from the same explant, at the same time, and under the same conditions, from two types of cells with different cytological (compact and friable) and morphogenic competencies (embryogenic and non-embryogenic) [17]. Therefore, these cell populations with distinct cell fates constitute a favorable system for fundamental research on the molecular mechanisms underlying embryogenic ability in woody species. By revealing the factors involved in the acquisition and expression of this competence, it will allow for the improvement of the plant regeneration process in economically relevant crops.

Here, a detailed methodology that combines poly(A) RNA isolation and barcoded cDNA library preparation is described for plant callus samples.

## 2. Experimental Design

### 2.1. Plant Material

**Figure 1:**
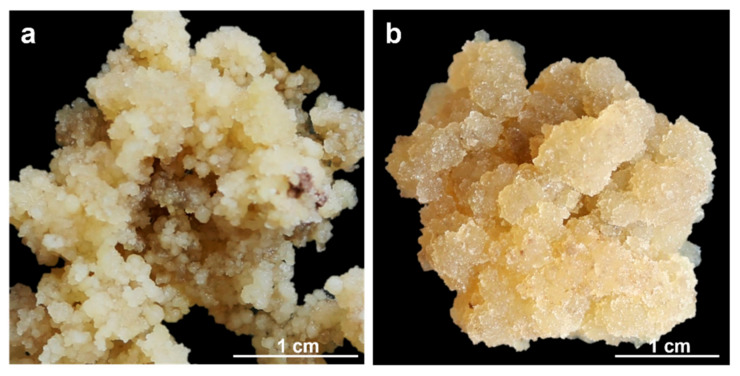
*S. betaceum* induced cell lines: (**a**) compact and embryogenic callus (EC); (**b**) friable and non-embryogenic callus (NEC).

Two *Solanum betaceum* Cav. cell lines were used: a compact embryogenic callus (EC, Figure 1a) and a friable non-embryogenic callus (NEC, Figure 1b). Both calluses were induced from leaf explants of in vitro growing micropropagated shoots, following the methodology previously described [18]. Callus lines cultures were maintained in Murashige and Skoog medium [19] (© Duchefa Biochemie, Haarlem, The Netherlands), supplemented with 20 μM Picloram (Sigma-Aldrich^®^, St. Louis, MO, USA) plus 9% (*w*/*v*) sucrose, solidified with 0.25% (*w*/*v*) Phytagel™ (Sigma-Aldrich^®^, St. Louis, MO, USA) at pH 5.7 and incubated at 24 ± 1 °C under dark conditions. For each callus line, EC, and NEC, four biological replicates were used.

### 2.2. Reagents

1. Agencourt AMPure XP beads (Beckman Coulter, Inc., Brea, CA， USA, EUA, Cat. No. A63880)
2. Blunt/TA Ligase Master Mix (New England Biolabs Inc. (NEB), Ipswich, MA, USA, EUA, Cat. No. M0367)
3. Direct cDNA Sequencing Kit (ONT, Oxford, UK, Cat. No. SQK-DCS109)
4. dNTPs NZYMix (NZYTech, Lda. (NZY), Lisbon, Portugal, Cat. No. MB086)
5. Dynabeads™ mRNA DIRECT^TM^ Kit (Invitrogen™, Thermo Fisher Scientific, Massachusetts, EUA, Cat. No. 61011)
6. Ethanol for molecular biology (Sigma-Aldrich^®^, Cat. No. 51976)
7. Flow Cell R9.4.1 (ONT, Cat. No. FLO-MIN106)
8. LongAmp^®^ Taq 2X Master Mix (NEB, Cat. No. M0287)
9. Maxima H Minus Reverse Transcriptase (Thermo Scientific™, ™, Thermo Fisher Scientific, Massachusetts, EUA, Cat. No. EP0751)
10. Native Barcoding Expansion 1–12 (ONT, Cat. No. EXP-NBD104)
11. NEBNext^®^ Quick Ligation Module (NEB, Cat. No. E6056)
12. NEBNext^®^ Ultra™ II End Repair/dA-Tailing Module (NEB, Cat. No. E7546)
13. Nuclease-free water (PanReac AppliChem ITW Reagents, Barcelona, Spain, Cat. No. A7398)
14. NZYRibonuclease Inhibitor (NZY, Cat. No. MB084)
15. Qubit^TM^ dsDNA HS Assay Kit (Invitrogen™, Cat. No. Q32851)
16. Qubit^TM^ RNA High Sensitivity (HS) Assay Kit (Invitrogen™, Cat. No. Q32852)
17. RNase Cleaner (NZY, Cat. No. MB16001)
18. RNase Cocktail™ Enzyme Mix (Invitrogen™, Cat. No. AM2286)

### 2.3. Supplies

1. 0.2 mL PCR tubes
2. 1.5 mL microcentrifuge tubes
3. 21-Gauge needles, sterile
4. Liquid nitrogen
5. Low-binding P10 barrier tips, sterile
6. Low-binding P1000 barrier tips, sterile
7. Low-binding P200 barrier tips, sterile
8. Mortars
9. Pestles
10. Qubit™ Assay Tubes (Invitrogen™, Cat. No. Q32856)
11. Spatulas
12. Sterilized syringes

### 2.4. Equipment

- DynaMag^TM^-2 magnet (Invitrogen™, Cat. No. 12321D)
- Heating block
- Microspin
- MinION (ONT, MK1C)
- NanoDrop™ 2000 spectrophotometer (Thermo Scientific™, Cat. No. ND-2000)
- Qubit^TM^ 4 fluorometer (Invitrogen™, Cat. No. Q33238)
- Rotator mixer, used at a rotation speed of ~25 rpm
- Thermo Scientific™ Arktik™ thermal cycler (Thermo Scientific™, Cat. No. 11999984)
- Vacuum concentrator
- Vortex mixer

### 2.5. Software

- MinKnow (ONT, version 21.10.8)

## 3. Procedure

The optimized steps in the protocol are indicated with the 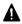 symbol.

### 3.1. Setup

Clean mortars, pestles, and spatulas with ethanol and RNase Cleaner before autoclaving.

Autoclave, twice, all no nuclease-free materials.

Work in an RNase-free environment, use RNase Cleaner and wear gloves.


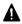
 Bring Agencourt AMPure XP beads to room temperature 30 min before use.

Prepare fresh 70% (*v*/*v*) ethanol before each Agencourt AMPure XP beads purification step.

### 3.2. Poly(A) RNA Purification

**Figure 2:**
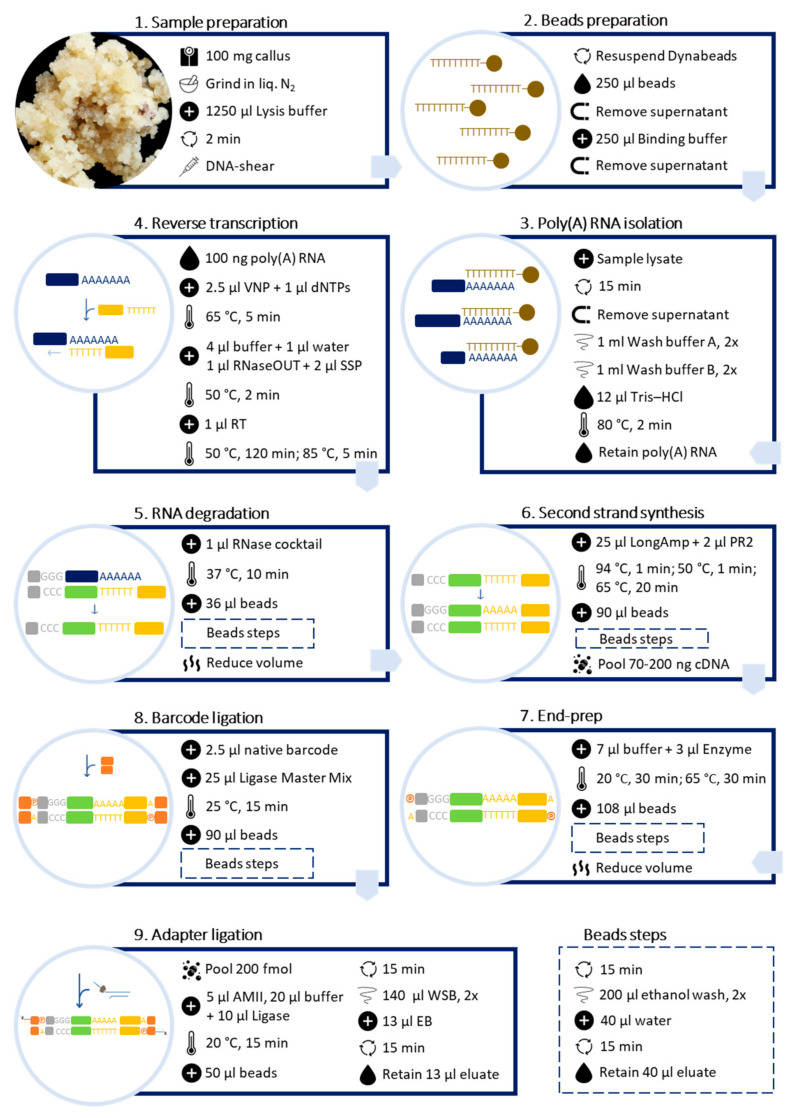
Schematic workflow for preparing barcoded cDNA libraries from plant callus for long-read sequencing, using Dynabeads™ mRNA DIRECT™ Kit (Invitrogen™) and Direct cDNA Sequencing Kit (ONT, Cat. No. SQK-DCS109) with Native Barcoding Expansion 1–12 (ONT, Cat. No. EXP-NBD104).

Poly(A) RNA was purified directly from callus samples using the Dynabeads™ mRNA DIRECT™ Kit (Invitrogen™) (Figure 2).

#### 3.2.1. Sample Preparation

1. Weigh 100 mg of fresh callus samples and flash-freeze in liquid nitrogen.
2. Grind the frozen material in liquid nitrogen, using a mortar and pestle, and transfer the fine powder to a microcentrifuge tube with a spatula.
  Maintain tubes in liquid nitrogen while all samples are processed.
3. Add 1250 µL of lysis/binding buffer and vortex for 2 min.
  To avoid the samples heating up, place the tubes on ice.
4. Force the lysate through a 21-gauge needle 3–5 times using a 1 mL syringe, to shear the DNA. Spin down briefly. This is a recommended step to avoid the beads clumping during incubation with sample lysate and mRNA contamination with DNA.

#### 3.2.2. Dynabeads™ Preparation

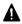
 Equilibrate lysis/binding buffer and wash buffers A and B to room temperature before use. Keep 10 mM Tris–HCl pH 7.5 (elution buffer) at 4 °C.
2. Thoroughly resuspend Dynabeads™ Oligo(dT)25.
3. Transfer 250 μL of the beads to a 1.5 mL microcentrifuge tube and place the tube on the DynaMag™-2 magnet.
4. When the suspension is clear, discard the supernatant.
5. Remove the tube from the magnet and wash the beads by resuspending them in 250 µL of fresh lysis/binding buffer.

#### 3.2.3. Poly(A) RNA Isolation

1. Place the washed beads on the magnet and discard the supernatant when the suspension is clear.
2. Remove the tube from the magnet and add the sample lysate from step 1.d., pipetting to resuspend the beads completely.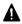 Incubate the mixture on a rotator mixer for 15 min at room temperature.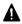 Place the tube on the magnet for 5 min. Rotate the tube slowly 90° to the right, and then, back to the starting position. Do the same for the left and hold for more than 5 min. When the suspension is clear, discard the supernatant.
5. Remove the tube from the magnet and wash the RNA-beads complex, twice, by pipetting 1 mL of washing buffer A until the beads are thoroughly resuspended.
  Use the magnet to completely remove all traces between each washing step.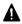 Wash the RNA-beads complex, twice, with 1 mL of washing buffer B by pipetting until the beads are thoroughly resuspended.
  Use the magnet to completely remove all traces between each washing step.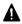 Elute the poly(A) RNA from the beads by adding 12 μL of 10 mM Tris–HCl (pH 7.5). Pipette until the beads are thoroughly resuspended, then, boil the tube at 80 °C for 2 min.
8. Immediately place the tube on the magnet and transfer the supernatant containing the poly(A) RNA to a new RNase-free tube. Place the poly(A) RNA tube on ice.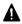 Analyze 1 μL of the poly(A) RNA using Qubit™ RNA High Sensitivity (HS) Assay Kit and 1 μL using a NanoDrop™ spectrophotometer.
  For cDNA synthesis, use only samples that present an A260/A280 ratio between 1.8 and 2.1 and an A260/A230 ratio higher than 1.8.

Barcoded libraries were prepared following the Direct cDNA Native Barcoding Nanopore protocol, available at https://store.nanoporetech.com/eu/direct-cdna-sequencing-kit.html (accessed on 23 February 2022, version: DCB_9091_v109_revN_14Aug2019), with modifications (Figure 2).

### 3.3. Reverse Transcription and Strand-Switching

1. Transfer 100 ng poly(A) RNA into a 0.2 mL PCR tube and adjust the volume to 7.5 μL with nuclease-free water.
2. Add 2.5 μL of the VN primer and 1 μL of dNTPs (10 mM).
3. Mix thoroughly by pipetting and centrifuge, briefly.
4. Incubate at 65 °C for 5 min before snap-cooling on a prechilled freezer block.
5. Prepare a master mix with the following reagents:
  - 4 μL 5x RT Buffer
  - 1 μL NZY ribonuclease inhibitor
  - 1 μL nuclease-free water
  - 2 μL strand-switching primer
6. Mix thoroughly by pipetting and centrifuge, briefly.
7. Add 8 μL of the master mix to the snap-cooled RNA. Mix the tube and, briefly, centrifuge.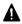 Incubate at 50 °C for 2 min.
9. Add 1 μL of Maxima H Minus Reverse Transcriptase. Gently mix the tube and, briefly, centrifuge.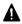 Incubate at 50 °C for 120 min and at 85 °C for 5 min. Then, maintain the tube at 4 °C.

### 3.4. RNA Degradation

1. Add 1 μL RNase Cocktail™ Enzyme Mix to the reverse transcription reaction.
2. Incubate the reaction at 37 °C for 10 min.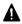 Vortex to resuspend the beads and transfer 36 μL to a new 1.5 mL microcentrifuge tube.
4. Add the reaction to the beads and mix thoroughly by pipetting.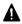 Incubate on a rotator mixer for 15 min at room temperature.
6. Centrifuge the sample and pellet with the magnet. Keeping the tube on the magnet, when the suspension is clear, remove the supernatant.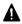 Keeping the tube on the magnet, wash the beads twice with 200 μL of freshly prepared 70% ethanol, without disturbing the pellet. After 1 min remove the ethanol with a pipette and discard.
8. Centrifuge, and replace the tube back on the magnet. Remove any residual ethanol and allow to air-dry for ~30 s; however, do not dry the pellet to the point of cracking.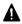 Remove the tube from the magnet and resuspend the pellet in 40 μL nuclease-free water.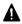 Incubate on a rotator mixer for 15 min at room temperature.
11. Pellet the beads with magnet until the eluate is clear and colorless.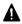 Remove and retain 40 μL of eluate in a new 1.5 mL microcentrifuge tube.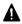 Allow the tubes to cool down, on ice, before transporting them, opened, to a vacuum concentrator to reduce the sample volume by up to 23 μL.

### 3.5. Second-Strand Synthesis

1. Transfer the samples to a 0.2 mL PCR tube and adjust the volume to 23 μL with nuclease-free water.
2. Add 25 μL of LongAmp^®^ Taq 2X Master Mix and 2 μL of the PR2 primer.
3. Mix thoroughly by pipetting and, briefly, centrifuge.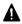 Incubate at 94 °C for 1 min, 50 °C for 1 min, and 65 °C for 20 min. Then, maintain the tube at 4 °C.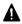 Vortex to resuspend the beads and transfer 90 μL to a new 1.5 mL microcentrifuge tube.
6. Repeat steps 4–12 from ‘RNA degradation’.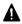 Analyze 1 μL of the strand-switched DNA using the Qubit^TM^ dsDNA HS Assay Kit from Invitrogen™.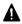 If necessary, pool cDNA samples to ensure 70–200 ng in a maximum volume of 50 μL. Use a vacuum concentrator to reduce the sample volume.

### 3.6. End-Prep

1. Transfer the samples to a 0.2 mL PCR tube and adjust the volume to 50 μL with nuclease-free water.
2. Add 7 μL of NEBNext Ultra II End Prep Reaction Buffer and 3 μL of NEBNext Ultra II End Prep Enzyme Mix.
3. Mix thoroughly by pipetting and, briefly, centrifuge.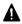 Incubate at 20 °C for 30 min and at 65 °C for 30 min.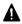 Vortex to resuspend the beads and transfer 108 μL to a new 1.5 mL microcentrifuge tube.
6. Repeat steps 4–12 from “RNA degradation”.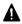 Allow the tubes to cool down on ice and transport them, opened, to a vacuum concentrator to reduce the sample volume by up to 22.5 μL.

### 3.7. Barcode Ligation

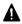
 Transfer Blunt/TA Ligase Master Mix to ice before the reaction setup. Mix the tube before use.
2. Adjust the sample volume to 22.5 μL with nuclease-free water.
3. Add 2.5 μL of a native barcode to each sample and 25 μL of Blunt/TA Ligase Master Mix.
4. Mix thoroughly by pipetting and, briefly, centrifuge.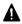 Incubate at 25 °C for 15 min.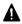 Vortex to resuspend the beads and transfer 90 μL to the reaction. Mix thoroughly by pipetting.
7. Repeat steps 5–12 from “RNA degradation”.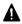 Analyze 1 μL of the eluted sample using Qubit™ dsDNA HS Assay Kit from Invitrogen™.
9. Estimate the average length of the cDNA sequences by electropherogram analysis (e.g., Agilent’s Bioanalyzer) or from previously obtained transcriptomic data analysis.

### 3.8. Adapter Ligation

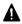
 Based on the predicted average length of the sequences and not exceeding 200 fmol, calculate the maximum cDNA concentration to use. For example, if the average length of sequences is 1000 bp, use a maximum of 130 ng.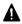 Calculate the volume to be used from each barcoded sample, and pool with the same concentration for all samples.
3. Pool the barcoded samples to get the maximum cDNA concentration in a 1.5 mL microcentrifuge tube. Adjust the volume to 65 μL by adding nuclease-free water or using a vacuum concentrator to reduce the sample volume.
4. Thaw Wash Buffer (WSB), Elution Buffer (EB), and NEBNext Quick Ligation Reaction Buffer (5X) at room temperature. Vortex to mix, centrifuge, and place on ice. Check the contents of each tube are clear of any precipitate. Centrifuge the T4 Ligase and the Adapter Mix II (AMII) and place them on ice.
5. Add 5 μL of Adapter Mix II (AMII), 20 μL of NEBNext Quick Ligation Reaction Buffer (5X), and 10 μL of Quick T4 DNA Ligase to the pooled and barcoded DNA, mixing the tube between each sequential addition.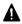 Centrifuge and incubate at 20 °C for 15 min.
7. Vortex to resuspend the beads and transfer 50 μL to the reaction.
8. Mix thoroughly by pipetting.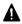 Incubate on a rotator mixer for 15 min at room temperature.
10. Pellet the beads with the magnet and, when the suspension is clear, remove the supernatant.
11. Wash beads–DNA complex twice with 140 μL of the Wash Buffer (WSB). Close the tube lid and resuspend the beads in the tube. Return the tube to the magnet, allow beads to pellet, and remove the supernatant.
12. Centrifuge and place the tube back on the magnet. Remove any residual supernatant.
13. Remove the tube from the magnet and resuspend the pellet in 13 μL Elution Buffer (EB).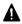 Incubate on a rotator mixer for 15 min at room temperature.
15. Pellet the beads with the magnet until the eluate is clear and colorless.
16. Remove and retain 13 μL of eluate into a clean 1.5 mL microcentrifuge tube.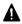 Quantify 1 μL of the eluted sample using Qubit™ dsDNA HS Assay Kit from Invitrogen™.
18. Fragment size and purity of cDNA libraries can be assessed using an Agilent Bioanalyzer High Sensitivity DNA chip.

### 3.9. ONT Sequencing

Perform priming and loading of the SpotON flow cell, and data acquisition and base-calling in real-time using MinION and MinKNOW, following, exactly, the described in the Direct cDNA Native Barcoding Nanopore protocol, available at https://store.nanoporetech.com/eu/direct-cdna-sequencing-kit.html (accessed on 23 February 2022, version: DCB_9091_v109_revN_14Aug2019).

### 3.10. Data Quality Assessment

Quality control of the raw data generated from each library can be verified by FastQC (or by a long-read specific QC tool like NanoQC). 

In the present work, data quality was assessed by FastQC (version 0.11.9) and a graph showing the Phred quality score per base sequence for all libraries was created using the MultiQC tool.

## 4. Results and Discussion

Aiming to perform ONT sequencing in difficult plant tissues, an optimized protocol for preparing barcoded cDNA libraries from callus samples with very distinct histological and morphogenic features was developed. The protocol combines high-yield and quality poly(A) RNA isolation and barcoded cDNA library preparation.

**Figure 3:**
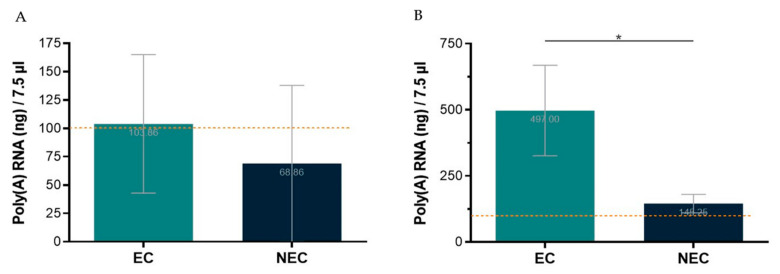
Poly(A) RNA isolation yield from embryogenic (EC) and non-embryogenic (NEC) callus samples, in the maximum volume allowed to proceed to library preparation protocol. The dotted line represents the minimum threshold (100 ng/7.5 µL) required for library preparation. (**A**)—Starting with 61–100 µg total RNA and using Dynabeads™ mRNA Purification Kit (Invitrogen™). (**B**)—Starting directly from ~100 mg of callus and using Dynabeads™ mRNA DIRECT^TM^ Kit (Invitrogen™). Results are presented as the mean of seven (**A**) or four (**B**) replicates ± SD. * indicates significant differences by *t*-test at *p* < 0.05.

The first tested protocol for poly(A) RNA isolation was performed from total RNA using the Dynabeads™ mRNA Purification Kit (Invitrogen^TM^). Total RNA from *S. betaceum* EC and NEC was extracted using NZYol (NZY, Cat. No. MB18501) and the Direct-zol™ RNA MicroPrep from the Zymo^©^ Research Corporation. As recommended by the manufacturer’s protocol, one to three samples were pooled to achieve starting material of 75 µg of total RNA. Nevertheless, even using a range of 61–100 µg of total RNA, with good NanoDrop™ quality ratios, and following the manufacturer’s protocol, it was only possible to obtain the required 100 ng of poly(A) RNA in 7.5 µL, for the Direct cDNA Native Barcoding protocol, in less than 50% of the NEC samples used (Figure 3A). Thus, this approach proved ineffective to reach the required poly(A) RNA concentration, entailing significant losses of tissue samples and reagents due to the need to pool total RNA extractions from three replicates of ~100 mg fresh callus samples. Therefore, another kit for poly(A) RNA isolation was used, the Dynabeads™ mRNA DIRECT™ Kit (Invitrogen™). With this kit, poly(A) RNA was isolated directly from callus samples, which enabled the required poly(A) RNA concentration to be achieved using much less tissue, extractions, and time (Figure 3B).

It is noteworthy that the poly(A) RNA yield for NEC is lower than for EC. Indeed, NEC is a friable watery callus with bigger and highly vacuolated cells compared to EC [17]. Therefore, it is expected that, from samples with the same fresh weight, lower yields of RNA are obtained. This is an aspect that deserves particular attention when optimizing such protocols for highly heterogenous samples/tissues. 

For the preparation of the cDNA libraries, the Direct cDNA Native Barcoding from the ONT protocol was initially followed. However, DNA losses were verified, which impaired the following steps. For instance, even starting with 100 ng/7.5 µL poly(A) RNA and following all the steps, as described, the cDNA concentration was around 0.6 ng/µL before the end-prep step, instead of at least 3.5 ng/µL, as recommended by ONT. In fact, several hard-to-remove contaminants inherent to plant tissues (e.g., phenolic compounds and polysaccharides) inhibit enzymes in library preparation processes for nucleic acid sequencing [11,13,20]. Therefore, optimizations were also needed. First, the incubation time was prolonged for all the reactions. Moreover, the incubation temperature of the Maxima H Minus Reverse Transcriptase (Thermo Scientific™) was 50 °C (not 42 °C, as suggested by the ONT protocol), since this was the optimal reaction temperature described for this enzyme by the manufacturer. Finally, to guarantee that all DNA was recovered in the Agencourt AMPure XP beads purification steps, adequate beads, and elution volumes were used. As recommended by the manufacturer’s protocol, 1.8 µL AMPure XP was added per 1.0 µL of the sample (almost twice the volume of the beads suggested by the ONT protocol). After washing with ethanol, the beads were always eluted in 40 µL, since, as indicated by the manufacturer, recovery decreases as the elution volume decreases. In several cases, to overcome the problem of too much volume, a vacuum concentrator was used. Altogether, only by extending the incubation time of all reactions, using the optimal incubation temperature for the reverse transcriptase, and using adequate beads and elution volumes, was the recommended cDNA concentration achieved for the end-prep step.

**Figure 4:**
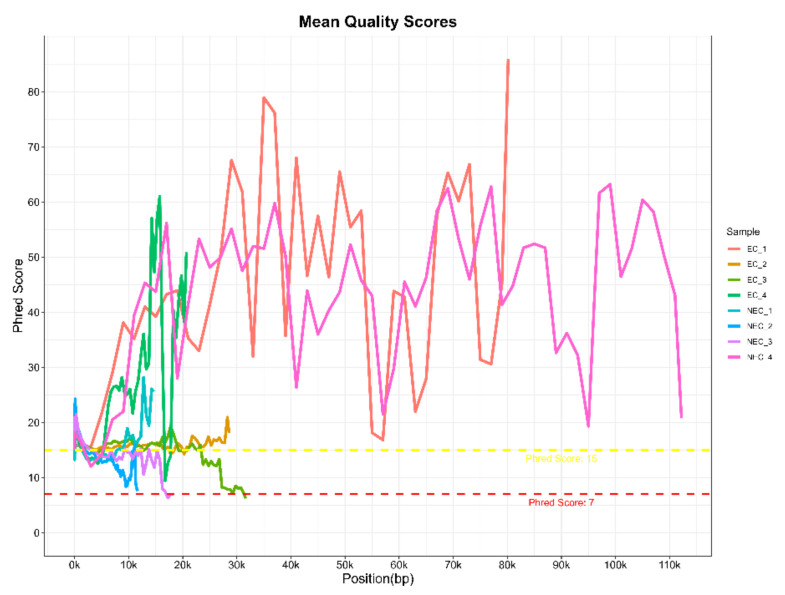
Raw data quality assessment of long-reads generated from barcoded cDNA libraries of *S. betaceum* compact embryogenic callus (EC) and friable non-embryogenic callus (NEC) by MinION sequencing from Oxford Nanopore Technologies^®^. The graph shows the Phred quality score per base sequence for all libraries, which was created with the MultiQC tool from the FastQC reports.

With this protocol, eight barcoded cDNA libraries from two *S. betaceum* cell lines, with different histological and embryogenic competencies, were prepared and sequenced. Libraries were pooled and direct cDNA sequencing was carried out using one flow cell in MinION equipment. In a single run, 1.78 M raw reads were generated in total, with an estimated average read length N50 of ~1.2 kb. Following the Nanopore protocol, only reads with a minimum q-score of eight (corresponding to a base call accuracy of >80%) were selected for further analysis. Nevertheless, to verify the quality of the raw data generated, FastQC reports were performed for each library (Table 1 and Supplementary Material Figure S1). Despite the low reads count, it is noteworthy that cDNA libraries were prepared only from polyadenylated transcripts, which comprise a smaller fraction of the total RNA. Moreover, Nanopore sequencing of plant samples often leads to higher pore inactivation, even when loading the allowed library concentration. Indeed, the samples used may have conditioned the flow cell occupancy rate, which was around 40–50% (Supplementary Material Figure S2), below the 70% threshold indicated by Nanopore. Nevertheless, it is an acceptable occupancy rate [21], without compromising the quality of the reads, as verified by the analysis of their quality. Despite the high dissimilarity in the Phred quality score per base sequence across all bases among the different libraries, a mean quality score over 15 was found (Figure 4). This indicates a base call accuracy higher than 96.8% [22], which is considered as assigning confidence for long-reads with little effect on downstream analysis [23,24]. Therefore, barcoded libraries prepared using this protocol are suitable for further analysis. 

## 5. Conclusions

An optimized and detailed protocol was developed, which combines poly(A) RNA isolation using Dynabeads™ mRNA DIRECT^TM^ Kit (Invitrogen™) and cDNA library preparation using Direct cDNA Sequencing Kit (ONT, Cat. No. SQK-DCS109) with Native Barcoding Expansion 1–12 (ONT, Cat. No. EXP-NBD104). Barcoded cDNA libraries prepared following this method can be used for long-read sequencing methods. Moreover, this protocol can be applied to other plant species, overcoming the recalcitrance of some plant tissues to these molecular biology methods, which remains a limitation of gene expression studies.

## Figures and Tables

**Table 1:** Basic statistics from FastQC reports of raw data generated by MinION sequencing from Oxford Nanopore Technologies^®^ from barcoded cDNA libraries of *S. betaceum* compact embryogenic callus (EC) and friable non-embryogenic callus (NEC).

Library	Number of Reads	Read Length Distribution (bp)	% GC
EC_1	256,799	1–80,322	38
EC_2	290,623	1–28,627	42
EC_3	369,635	1–31,788	45
EC_4	120,876	1–20,839	35
NEC_1	166,753	1–14,734	42
NEC_2	134,307	1–11,778	41
NEC_3	203,020	1–18,416	39
NEC_4	236,832	1–112,383	39

## Data Availability

The datasets generated during the current study are available in the NCBI repository with the submission ID SUB12178214 (BioProject ID PRJNA892465; Biosample Accessions SAMN31382762 and SAMN31382764).

## Supplementary Materials

The following supporting information can be downloaded at: https://www.mdpi.com/article/10.3390/mps6020031/s1, Figure S1: Sequence length distribution, Figure S2: Duty time plot.
