# Supplementary material for: An Efficient Method to Prepare Barcoded cDNA Libraries from Plant Callus for Long-Read Sequencing
Source: Methods Protoc. 2023 Mar 15;6(2):31. doi: 10.3390/mps6020031 (PMC10037601; doi:10.3390/mps6020031)
Supplement: Supplementary file 1 [file mps-06-00031-s001.zip › mps-2219963-supplementary.pdf]

Sequence length distribution

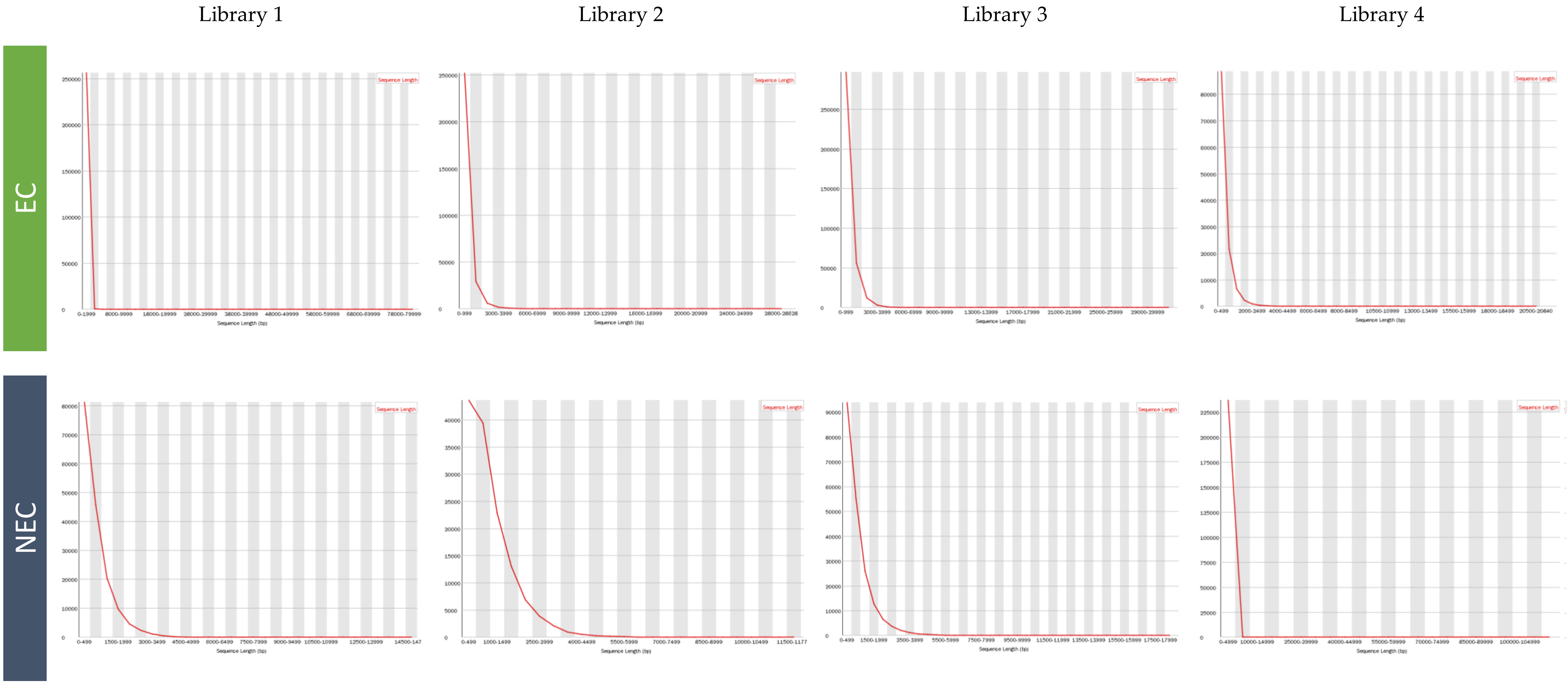

Figure S1. Distribution of sequence lengths, over all sequences of long-reads, generated from barcoded cDNA libraries, of *S. betaceum* compact embryogenic callus (EC) and friable non-embryogenic callus (NEC), by MinION sequencing from Oxford Nanopore Technologies®.

Duty time gruped

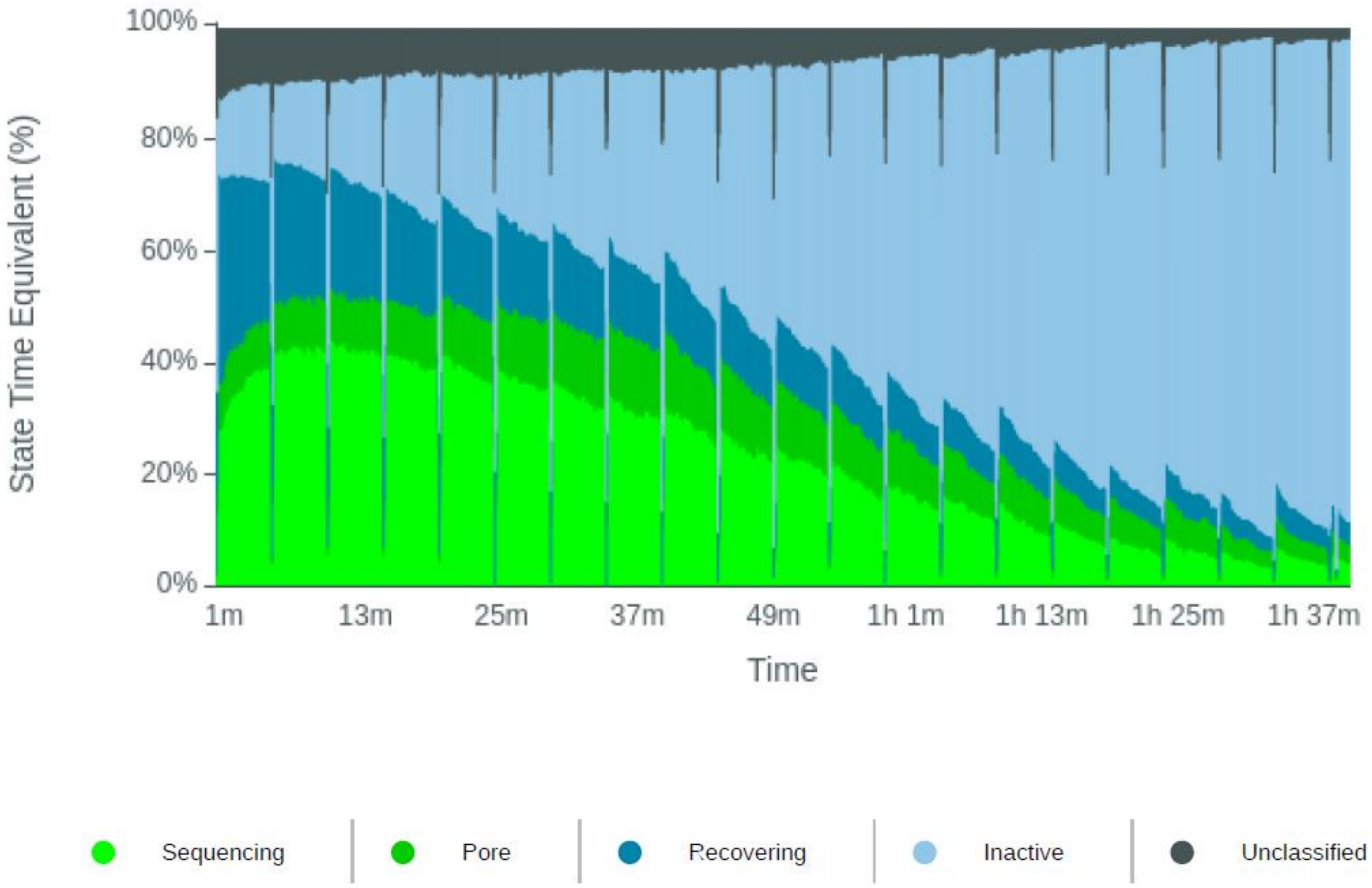

Figure S2. Duty time plot, obtained by MinKNOW to access the quality of run carried out by MinION sequencing from Oxford Nanopore Technologies®.
